# The Impact of Smoking, Alcohol Use, Recurrent Disease, and Age on the Development of Neck Fibrosis in Head and Neck Cancer Patients Following Radiation Therapy

**DOI:** 10.3389/fonc.2021.707418

**Published:** 2021-08-13

**Authors:** Connor L. Pratson, Michael C. Larkins, Brandon H. Karimian, Caitrin M. Curtis, Pamela A. Lepera, Brian N. Brodish, Andrew W. Ju

**Affiliations:** ^1^Brody School of Medicine, East Carolina University, Greenville, NC, United States; ^2^Division of Hematology/Oncology, Department of Internal Medicine, Brody School of Medicine, East Carolina University, Greenville, NC, United States; ^3^Eastern Carolina Head and Neck Surgery, Greenville, NC, United States; ^4^Department of Radiation Oncology, Brody School of Medicine, East Carolina University, Greenville, NC, United States

**Keywords:** head and neck cancer, fibrosis, radiotherapy, alcohol and tobacco use, risk factors

## Abstract

There is a paucity of information regarding the demographic factors associated with the development of neck fibrosis in head and neck cancer (HNC) patients following radiotherapy. A retrospective review of all patients being treated for HNC at a tertiary care center between 2013 and 2017 was performed. Chi-squared and Mann-Whitney U tests were used to identify differences in incidence and grade of fibrosis, respectively, between populations. A total of 90 patients aged 19 to 99 years were included. Factors associated with an increased incidence of fibrosis included smoking during radiotherapy (*p* < 0.001), alcohol use (*p* = 0.026), recurrent disease (*p* = 0.042), and age less than 60 (*p* < 0.001) on univariate analysis. Factors associated with increased grade of fibrosis in HNC patients included recurrent HNC (*p* = 0.033), alcohol use (*p* = 0.013), patient age younger than 60 years (*p* = 0.018), smoking during radiotherapy (*p* < 0.001), and non-Caucasian race (*p =* 0.012). Identification and intervention directed at patients that possess risk factors associated with fibrosis prior to treatment has the potential to improve the long-term quality of life for HNC patients.

## Introduction

Head and neck cancers (HNC) carry a significant disease burden, accounting for 3% of all cancers in the United States. More than 53,000 Americans are diagnosed with HNC each year, with approximately 10,800 deaths (1). Although the overall incidence of HNC is decreasing in the US (2), the incidence of human papilloma virus (HPV)-associated cancers, especially of the oropharynx, is increasing (2, 3). Recent studies suggest HPV may account for about 70 to 80% of oropharyngeal cancer in North America and Europe and may even confer a survival advantage (3). As medical advances continue to improve treatment of HNC, the population of HNC patients continues to increase. Thus, quality of life in HNC patients becomes a topic of increasing importance.

The side effects of chemotherapy, radiotherapy, and/or surgery can significantly affect the quality of life of HNC survivors; examples common in radiotherapy include trismus, xerostomia, dermatitis, dysphagia, and radiation fibrosis (4). Radiation fibrosis is commonly described as a late complication of HNC treatment modalities that may not manifest clinically for several months or years after treatment. Fibrosis impacts quality of life due to associated pain, sensory loss, myopathy, dysphagia, trismus, and limitation in range of motion, impacting patients’ ability to perform self-care, speak, eat, etc. (5). Current potential treatments used for fibrosis secondary to radiation therapy include pentoxifylline, vitamin E, and Low-Level Light Therapy (LLLT); devices designed to aid with jaw strength rehabilitation following the development of trismus also exist (6).

Treatment factors such as history of previous neck dissection, concurrent chemotherapy, corticosteroid administration, and dosage/fraction size of radiation have been identified as risk factors for fibrosis development (7). However, variation in radiosensitivity is observed even amongst patients undergoing the same treatment regimen. There is limited data on the demographic risk factors associated with the specific development of fibrosis in HNC patients following radiotherapy. Identification of at-risk patients prior to or early in the treatment process can help providers address potential side-effects prior to symptomatic manifestation and help patients prepare for functional changes. Furthermore, helping patients address modifiable risk factors associated with fibrosis can decrease the incidence and severity of fibrosis (e.g. informing patients with a history of tobacco use that this history may contribute to increased risk of neck fibrosis development peri- or post-therapy). Early intervention, such as with smoking/alcohol cessation, has the potential to improve patient outcomes.

## Materials and Methods

### Data Collection

At a tertiary care hospital serving a largely rural region, patients with a diagnosis of cancer of the head and neck from the years 2013 through 2017 were identified. Exclusion criteria included age under 18 years old at time of initiation of treatment, patients being treated without curative intent, death before completion of radiotherapy, and non-primary head and neck cancers. Patients lost to follow-up and those with insufficient follow-up in the 6-month post-treatment period were also excluded from the final data analysis.

Retrospective data were collected from the patients’ electronic medical records. Demographic data were collected regarding potential risk factors for fibrosis including race, socioeconomic status (as defined by insurance coverage), use of tobacco, consumption of alcohol, age, and treatment of recurrent disease. Other data collected included tumor stage according to 8^th^ edition AJCC when possible (though AJCC 7^th^ edition was utilized for some patients when HPV status was unknown, 8, 9), radiotherapy modalities used, overall survival, and incidence/severity of radiation related toxicities. The Common Terminology Criteria for Adverse Events (CTCAE) version 4.0 was used to grade the severity of radiation-related toxicities (10). According to this terminology, which ranges from Grades 1 to 5, Grade 1 refers to an AE (Adverse Event, any unfavorable sign such as an abnormal lab finding) that is mild/asymptomatic. A Grade 2 AE is “moderate; minimal, local, or noninvasive intervention indicated.” A Grade 3 AE is “severe or medically significant but not immediately life-threatening…” A Grade 4 AE is life-threatening and a Grade 5 AE results in death.

Determination of neck fibrosis was determined clinically by treating physicians, from specialties including otolaryngology, medical oncology, and radiation oncology according to CTCAE and NCCN guidelines (10). According to CTCAE guidelines Grade 1 soft tissue fibrosis consists of mild induration, with patient able to move skin parallel and perpendicular to the tissue plane. Grade 2 consist of moderate induration with the skin unable to be pinched. Grade 3 consists of serve induration with inability to slide or pinch skin. Grade 4 consists of generalized symptoms including impaired breathing or feeding and Grade 5 consists of death.

### Study Population

A total of 141 HNC patients were identified. A primary neoplasm found outside of the head and neck excluded 16 patients. There were 24 patients lost to follow up, and 6 patients died during treatment. Initially a noncurative intent of the treatment excluded 5 patients due to no expectation of long-term follow-up, and 2 patients were less than 18 years old. A total of 90 patients (**Table 1**) were included in the final study cohort, including 62 males and 28 females with a median age of 62. African Americans made up 49% of the study population, while Caucasians comprised 49%. Tumor Stages I to IVC were considered for treatment in the final patient cohort. Patients with onset of neck fibrosis less than 6 months after treatment were considered acute, and greater than 6 months were considered delayed.

**Table 1:** Patient demographics, tumor characteristics, and treatment characteristics of the study population (N=90).

Characteristics	# of Patients (%)
**Median Age**	62 years (range: 19-99 years)
**Gender**	
Male	62 (69%)
Female	28 (31%)
**Race**	
African American	44 (49%)
Caucasian	44 (49%)
Hispanic	1 (1%)
Other	1 (1%)
**Insurance Coverage**	
Private	14 (16%)
Medicare + supplemental	17 (19%)
Medicare only	29 (32%)
Medicaid	29 (32%)
Uninsured	1 (1%)
**Primary Site of Tumor**	
Larynx/hypopharynx	32 (36%)
Oropharynx	27 (30%)
HPV positive	9 (10%)
HPV negative	5 (6%)
HPV unknown	13 (14%)
Oral cavity	6 (7%)
Nasopharynx	12 (13%)
Salivary gland	6 (7%)
Thyroid	1 (1%)
Other	6 (7%)
**HPV Status (overall cohort)**	
HPV positive	15 (17%)
HPV negative	16 (18%)
HPV unknown	59 (65%)
**Overall Stage**	
I	8 (9%)
II	12 (13%)
III	16 (18%)
IVA	45 (50%)
IVB	8 (9%)
IVC	1 (1%)
**Histology**	
Squamous	77 (86%)
Other	13 (14%)
**Treatment of Recurrent Cancer**	
No	79 (82%)
Yes	11 (12%)
**Prior Head and Neck Radiation**	
No	84 (93%)
Yes	6 (7%)
**Treatment modality**	
Radiation alone	7 (8%)
Chemotherapy and radiation	38 (42%)
Surgery and radiation	10 (11%)
Surgery, chemotherapy, radiation	35 (39%)

All patients included in this study were treated for primary HNC with radiotherapy. Half of the 90 patients included in the final study had Stage IVA tumors (**Table 1**). One patient had an overall tumor Stage of IVC and was treated with curative intent for a primary neoplasm on the nasopharynx. Of the 11 patients with treatment for recurrent cancer, 6 of these patients (55% of those with recurrent cancer) had received prior HNC radiation (7% of the total patient cohort).

### Treatment Details

4 patients were treated palliatively (patients were included in study due to expected to have adequate follow-up), with doses between 30 - 37.5 Gy at 10-15 fractionations; all others were treated with adjuvant or definitive doses between 48 Gy at 20 fractionations and 75 Gy at 60 fractionations, at daily doses of about 1.8 to 2.4 Gy. Dosage to organs at risk for all definitive cases. All definitive patients had dosing to organs at risk *via* IMRT with IGRT. With regards to potential tissue fibrosis, all treatments were done within QUANTEC/RTOG constraints; the dose to soft tissues was not specifically contoured. Not all dose metrics for muscular constrictors or pharyngeal muscles are retrievable due to limited archive space.

### Data Analysis

MedCalc V12.6 statistical software (Ostend, Belgium) was used for data analysis. Chi-squared and Mann-Whitney U tests were used to identify differences in incidence and grade of fibrosis, respectively, between populations. Logistic regression analysis was used to further evaluate the impact of these factors on the development of fibrosis. Logistic variables assessed include smoking versus non-smoking during radiotherapy, patient positive for alcohol use *versus* negative for alcohol use during radiotherapy, tumor Stage less than 4 *versus* Stage greater than or equal to 4, tumor recurrence *versus* no recurrence, patient age less than 60 years *versus* greater than 60 years, patient race being Caucasian *versus* non-Caucasian, and patient being on Medicaid or uninsured *versus* having other insurance; continuous variables (e.g. age) were converted into binary values *via* grouping. Multivariate analysis was carried out across all patients to exclude confounding variables such as radiotherapy using as an adjunct to chemotherapy or surgery (refer to **Table 1** for full patient cohort characteristics). P values < 0.05 were the threshold used to determine significance.

## Results

### Incidence of Neck Fibrosis

Following radiation therapy, 5 patients developed acute neck fibrosis (onset less than 6 months after radiotherapy) and 9 developed delayed neck fibrosis (duration greater than 6 months after radiotherapy); only one patient with acute symptoms went on the develop delayed fibrosis. Regarding CTCAE neck fibrosis Grade, 7 patients developed Grade 1 neck fibrosis and 4 developed Grade 2 neck fibrosis, from the final patient population of 90; no other Grades of neck fibrosis were observed. Factors associated with increased incidence of fibrosis are shown in **Table 2**. On univariate analysis, active smoking at the time of radiation oncology consultation was associated with an increased incidence of fibrosis (*p* < 0.001). This was affirmed by multivariate analysis, which showed a logistic regression *p*-value of less than 0.001. Active alcohol use during treatment was associated with an increased incidence of fibrosis on univariate analysis (*p* = 0.026) but was not found to be significantly associated on multivariate analysis. Treatment of recurrent disease was found to be significantly associated on univariate analysis (*p* = 0.042) but was not retained on multiple logistic regression. Patient age less than 60 years was significantly associated with incidence of fibrosis on univariate analysis (*p* < 0.001) but was not retained on multiple logistic regression.

**Table 2:** Demographics present during treatment contributed to incidence of fibrosis.

Demographic	Fibrosis	No Fibrosis	Univariate *p*-value	Logistic Multivariate *p*-value
Active Smoker Nonsmoker	8 3	15 64	<0.001*	<0.001*
Active Drinker Non-drinker	6 5	15 64	0.026*	NR
Stage < 4 Stage ≥ 4	35 44	2 9	0.19	NR
Tumor recurrence No tumor recurrence	7 4	22 57	0.042*	NR
Age < 60 Age > 60	11 0	50 29	<0.001*	NR
Caucasian Non-Caucasian	2 9	42 37	0.06	NR
Medicaid/uninsured Other insurance	6 5	24 55	0.21	NR

P-values are reported as derived from univariate analysis (chi-squared) and logistic multivariate analysis. (N = 90) * = significant. NR, not retained. Smoking and alcohol refers to active use during therapy.

On univariate analysis, overall Stage at diagnosis and insurance type were not associated with the development of fibrosis (*p* = 0.19 and 0.21, respectively, see **Table 2**). No statistical significance was found for patients to develop greater than or equal to Stage 4 fibrosis vs for patients to develop less than Stage 4 fibrosis. The impact of race on fibrosis approached significance for non-Caucasian patients developing fibrosis vs Caucasian patients on univariate analysis (*p* = 0.06) but did not meet the authors’ cutoff for significance.

### Severity of Fibrosis

**Figure 1:**
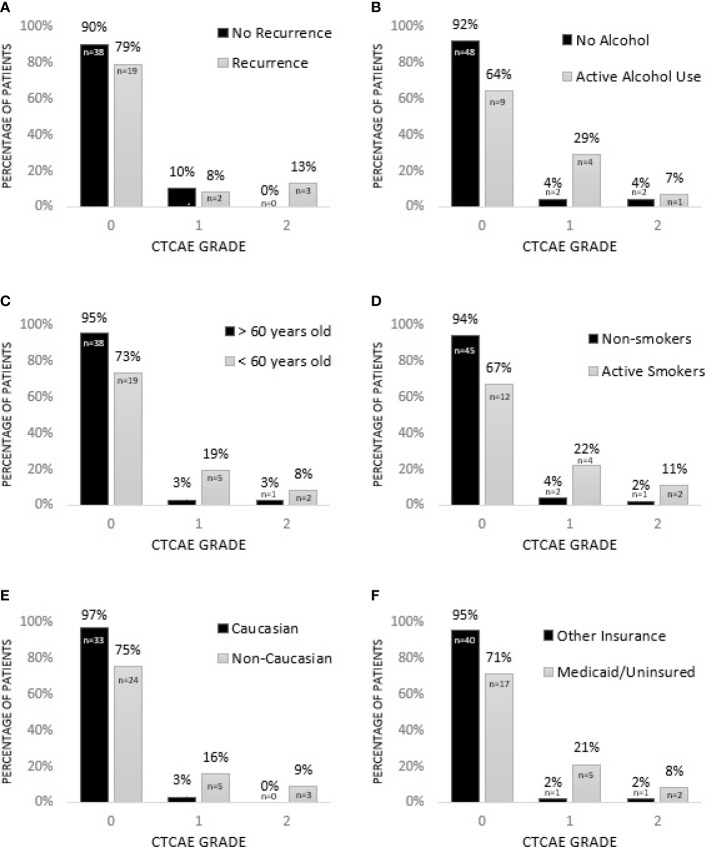
Factors associated with increased grade of fibrosis in HNC patients treated with radiotherapy. **(A)** Patients being treated for recurrent HNC experience higher grade of fibrosis (*p* = 0.033*). **(B)** Consumption of alcohol during treatment is associated with higher grade of fibrosis (*p* = 0.013*). **(C)** HNC patients younger than 60 experience higher grade of fibrosis (*p* = 0.018*). **(D)** Smoking during treatment is associated with increased grade of fibrosis (*p* = < 0.001*). **(E)** Non-Caucasian race is associated with increased grade of fibrosis (*p* = 0.012*). **(F)** Grade of fibrosis in uninsured patients or patients with Medicaid approaches significance (*p* = 0.054). * = significant.

Factors associated with increased grade of fibrosis in HNC patients treated with radiotherapy (**Figure 1**) included patients being treated for recurrent HNC (*p* = 0.033), consumption of alcohol during treatment (*p* = 0.013), patient age younger than 60 (*p* = 0.018), smoking during treatment (*p* = <0.001) and non-Caucasian race (*p* = 0.012). A higher grade of fibrosis in uninsured patients or patients with Medicaid approached significance (*p* = 0.054).

### Incidences Besides Fibrosis

In addition to fibrosis, CTCAE concerning patient dysphagia, xerostomia, and dermatitis were recorded at regular intervals during patient follow up (**Table 3**). In the first 4 weeks, 8% of patients had at least 1 dysphagia CTCAE, 28% had at least one xerostomia CTCAE, and 42% had at least one dermatitis CTCAE. Eleven to 13 months later, these numbers resolved to 8% continuing to have dysphagia, 24% having xerostomia, and 0% having dermatitis.

**Table 3:** Number of patients with reported Common Terminology Criteria for Adverse Events (CTCAE) besides fibrosis between 0 weeks and 13 months after radiation therapy. N = 90.

Symptom	Patients (%) with CTCAE Symptom at 0 - 4 Weeks	Patients (%) with CTCAE Symptom at 5 - 15 Weeks	Patients (%) with CTCAE Symptom at 8 - 10 Months	Patients (%) with CTCAE Symptom at 11 - 13 Months
Dysphagia	7 (8%)	6 (7%)	7 (8%)	7 (8%)
Xerostomia	25 (28%)	28 (31%)	25 (28%)	22 (24%)
Dermatitis	38 (42%)	7 (8%)	0	0

## Discussion

Radiotherapy plays a vital role in the multimodal treatment of patients with HNC: approximately 80% of patients receive radiation at least once during their disease course (11). Fibrosis following radiotherapy is a well-described phenomenon, although the pathogenesis behind this late reaction has not been fully elucidated. One of the proposed mechanisms is that radiation-induced maladaptive inflammation (from reactive oxygen and nitrogen species) leads to a misguided wound healing response; this response is characterized by increased collagen deposition (from increased differentiation of fibroblasts to myofibroblasts), poor vascularization, and scarring (12). The most important driver of this response is the inflammatory signalling molecule transforming growth factor-β, which drives the conversion of fibroblasts into myofibroblasts (12). Because of the potential association of inflammation and fibrosis, it stands to reason that factors related to increased or ongoing inflammation during and immediately after treatment may increase the likelihood and/or severity of patients developing fibrosis.

The findings reported in this paper seem to indicate that ongoing tobacco and alcohol use during treatment both appear to separately contribute to the development of fibrosis in the observed population following head and neck radiotherapy. Tobacco smoke has been found to be an irritant, which can cause an increase in the severity and duration of inflammation, and additionally may delay the healing process, which may be related to tobacco smoke toxicity (13). Additionally, it has been theorized that smoking during cancer therapy may reduce the available oxygen with patients’ bodies, limited the effects of reactive oxygen species to destroy cancer cells and preventing patients’ bodies from adapting to cancer therapy readily (14). Both tobacco and alcohol, especially when used concurrently, have been seen to increase HNC overall; a multiplicative effect between tobacco and alcohol use in the development of HNC has been cited in literature. It has been proposed that alcohol, in addition to its carcinogenic properties, could act as a solvent for the carcinogens present in cigarette smoke, thus delivering a greater concentration of carcinogens to susceptible mucosa (15). It is particularly problematic, considering that research has shown that nearly half of smokers found to have oral or oropharyngeal cancer will continue to smoke after their diagnosis (16). However, tobacco cessation programs during HNC treatment have proven effective in reducing smoking and could be a useful resource for many patients (17). Emphasis on cessation during and after radiotherapy could be associated with an increased quality-of-life. Further studies regarding the impact of smoking cessation during head and neck cancer treatment are needed.

The results of this study seem to indicate that patients younger than 60 years of age at the time of diagnosis appear to have a higher risk for developing neck fibrosis. Radiation-induced fibrosis has been recently theorized to develop due to aging and exposure to toxins, *via* the mechanism of increased stem cell senescence from replicative or stress-related factors (18); however, there is a dearth of literature specific to the development of fibrosis post-radiation therapy with respect to patient age. Although the authors are unsure of the etiology of increased neck fibrosis in younger patients present in this study, it is hypothesized that more vigorous inflammatory responses in the robust immune systems of younger patients during radiation treatment may lead to more extensive post-radiation remodelling. The potential correlation of age and fibrotic response provides an interesting direction of future research in patient populations with different demographics and forms of cancer.

On univariate analysis, ethnicity and insurance impact the Grade but not the incidence of neck fibrosis. Although not robust independent risk factors, they may still aid in identifying an at-risk population. It has been shown in some studies that black patients are more likely than Caucasian patients to be diagnosed at a young age with HNC, in addition to rural patients being less likely to be diagnosed with HNC at a young age (19). Further research is needed to confirm the trends seen in this study across a larger population.

A limitation of this study is that it is retrospective in nature and the study population was limited to 90 patients. Furthermore, there was a variation of treatment modalities in this patient population (surgery, radiotherapy, and/or chemotherapy), though radiotherapy was the common denominator in all subjects. Further research is needed to confirm these trends across a large population and to determine the possible mechanisms by which age, tobacco, and alcohol use may be associated with fibrosis. Additionally, the small sample size of patients with Grade 1 and 2 neck fibrosis may limit the statistical analysis of factors which related to the severity of neck fibrosis.

In summary, the data reported in this paper revealed several patient-related factors that are associated with the development of fibrosis in HNC patients following radiotherapy. Although chemotherapy, radiation, and surgery all remain the cornerstones of treatment of HNC, recent developments in the field such as immunomodulators and radiation dose de-escalation have allowed for improved post-treatment quality of life while still maintaining high therapeutic efficacy (20, 21). These new approaches along with pre-treatment identification of those at risk for radiation-related toxicities such as fibrosis could help reduce the incidence and severity of these toxicities. Ultimately, addressing the identified risk factors for fibrosis in HNC patients has the potential to improve patients’ quality of life.

## Data Availability Statement

The raw data supporting the conclusions of this article will be made available by the authors, without undue reservation.

## Ethics Statement

The studies involving human participants were reviewed and approved by the East Carolina University and Medical Center IRB (UMCIRB) prior to its start and can be found under identification number 17-001039 and title: “Impact of socioeconomics on outcomes of head and neck cancers.” It is active as of the time of submission. Written informed consent for participation was not required for this study in accordance with the national legislation and the institutional requirements.

## Author Contributions

CP was the student investigator for this project, collected the data used in this paper, and wrote the first draft. ML finished compiling this paper, aided with statistical analysis, and submitted this paper for publication. BK aided with data analysis and corrected this paper. CC aided with table and figure creation and data analysis. BB and PL reviewed the analysis and provided comments. AJ served as the principal investigator on this project and completed statistical analysis of the gathered data. All authors contributed to the article and approved the submitted version.

## Funding

Funding for this project came from the East Carolina University (ECU) Brody School of Medicine Summer Scholars Program in 2017; $1400 was awarded to the first author CP. Additionally, another $1400 was provided to CP by the ECU Radiation Oncology Department (ROD) also for the study. CP was granted $60 by the ECU ROD in 2019 to submit an abstract to the 2019 ASTRO Annual Meeting, and additionally $275 was awarded to CP to aid with registration for the 2018 Head & Neck Symposium.

## Conflict of Interest

The authors declare that the research was conducted in the absence of any commercial or financial relationships that could be construed as a potential conflict of interest.

## Publisher’s Note

All claims expressed in this article are solely those of the authors and do not necessarily represent those of their affiliated organizations, or those of the publisher, the editors and the reviewers. Any product that may be evaluated in this article, or claim that may be made by its manufacturer, is not guaranteed or endorsed by the publisher.
